# Hermetic Packaging Based on Cu–Sn and Au–Au Dual Bonding for High-Temperature Graphene Pressure Sensor

**DOI:** 10.3390/mi13081191

**Published:** 2022-07-28

**Authors:** Junqiang Wang, Haikun Zhang, Xuwen Chen, Mengwei Li

**Affiliations:** 1 Academy for Advanced Interdisciplinary Research, North University of China, Taiyuan 030051, China; zhanghaikun0312@163.com (H.Z.); nuc_chenxuwen@163.com (X.C.); 2 Notional Key Laboratory of Instrumentation Science & Dynamic Measurement, North University of China, Taiyuan 030051, China

**Keywords:** Cu–Sn bonding, Au–Au bonding, graphene, high-temperature pressure sensor

## Abstract

A chip-level hermetic package for a high-temperature graphene pressure sensor was investigated. The silicon cap, chip and substrate were stacked by Cu–Sn and Au–Au bonding to enable wide-range measurements while guaranteeing a high hermetic package. Prior to bonding, the sample was treated with Ar (5% H_2_) plasma. The Cu–Sn bonding was firstly performed at 260 °C for 15 min with a pressure of 9.9 MPa, and the corresponding process conditions for Au–Au bonding has increased to 300 °C, 20 min and 19.8 MPa respectively. The average shearing strength was 14.3 MPa, and an excellent leak rate of 1.72 × 10^−4^ Pa·cm^3^/s was also achieved. After high-temperature storage (HTS) at 350 °C for 10 h, the resistance of graphene decreased slightly because the dual bonding provided oxygen-free environment for graphene. The leakage rate of the device slightly increased to 2.1 × 10^−4^ Pa·cm^3^/s, and the average shear strength just decreased to 13.5 MPa. Finally, under the pressure range of 0–100 MPa, the graphene pressure sensor exhibited a high average sensitivity of 3.11 Ω/MPa. In conclusion, the dual bonding that combined Cu–Sn and Au–Au is extremely suitable for hermetic packaging in high-temperature graphene pressure sensors.

## 1. Introduction

Graphene is a hexagonal, honeycomb-shaped, two-dimensional material with thickness of approximately 0.335 nm. It exhibits excellent properties and has been widely used in pressure tests, energy storage, photoelectric detection, biomedicine, heat dissipation and other fields [1,2,3,4,5,6]. It also shows great potential in flame retardant materials and medication testing [7,8]. Compared with other two-dimensional materials, the preparation process of a carbon nanotube pressure sensor is complicated, which will cause a poor compatibility with micro-nano manufacturing [9], MoS_2_ with low modulus (≈300 GPa) has better flexibility than graphene, but it is difficult to grow large-area and high-quality nanofilm [10]. The overall performance of graphene is superior; high carrier mobility (up to 200,000 cm^2^ V·s), Young’s modulus (about 1 TPa) and high temperature resistance (up to 2000 °C) [11,12,13] make it have greater potential in the field of high-temperature pressure sensors. In the past few years, the use of graphene in pressure sensors is gaining momentum. Using single and multilayer graphene sheets, Bunch et al. manufactured the first prototype of a suspended graphene pressure sensor [14]. Sorkin and Zhang studied the mechanical failure of pressure sensors based on graphene nanoflake using atomistic approach. The sensors consist of graphene films suspended over a SiC substrate [15]. Smith et al. demonstrated the piezoresistive effect in graphene while proposing a novel pressure sensor based on a suspended graphene membrane [16,17]. Wang et al. developed a suspended graphene pressure sensor whose sensing unit consisted of porous graphene film arrays [18].

Although graphene pressure sensors are developing rapidly, the suspended or SiN_x_-film structure limited pressure range. Previous graphene pressure sensors had no packaging, limiting its high-temperature applications. In our previous research, we fabricated a thin-film-structured graphene pressure sensor using Cu–Sn flip-chip bonding for hermeticity packaging that exhibits high temperature stability [19]. However, this thin-film-structure is relatively suitable for a pressure range lower than 1 MPa, and in the face of the requirements of a wide pressure range, it is necessary to develop a new structure.

Packaging pressure sensors are primarily used to realize sealing and electrical interconnection. This can be realized through different bonding methods, such as anode bonding, solder bonding, metal-to-metal direct bonding, eutectic bonding, and so on. Anode bonding is usually realized under conditions of high pressure and voltage (1000 V) [20]. These conditions can result in the fracture of graphene devices or the breakdown of graphene. Solder bonding is a low-cost, simple process, but it is required to print a wide solder to achieve excellent sealing. This bonding method is not conducive to reducing packaging size and realizes the miniaturization of pressure sensors [21,22,23]. Common eutectic bonding includes Cu–Sn and Au–Sn bonding, but the melting point of AuSn20, a eutectic alloy of Au and Sn, is only 278 °C, which obviously cannot meet the needs of high-temperature packaging. Direct metal bonding includes Cu–Cu and Au–Au bonding, etc. The oxidation problem of Cu–Cu bonding limits its development, while Au is a chemically inert substance with stable properties and high melting point, so Au–Au bonding is more suitable for high temperature packages. Moreover, metal-to-metal direct bonding and eutectic bonding is characterized by significantly high hermeticity; the stack structure can effectively realize the requirements of miniaturization of devices. This is almost suitable for realizing pressure cavity construction in all pressure sensors [24,25,26,27,28].

In this paper, a dual-bonding of Cu–Sn and Au–Au is proposed for wide-range graphene pressure sensor. The high hermetic package provided effective protection for graphene, and the three-layer stack structure ensured a wide pressure range. The thickness, structure, and layout of the bonding material were designed to obtain a stable interface. The excellent performance of dual bonding is proved by many tests. This bonding method helped realize high-hermeticity sealing connection in a graphene pressure sensor. Excellent high-temperature reliability was achieved, which helped in the integration of multilayer devices.

## 2. Materials and Methods

There are three steps to realize dual bonding (Cu–Sn and Au–Au) in the chip-level high-temperature graphene pressure sensors: test vehicle design, vehicle fabrication, and Cu–Sn and Au–Au Bonding.

### 2.1. Test Vehicle Design

**Figure 1:**
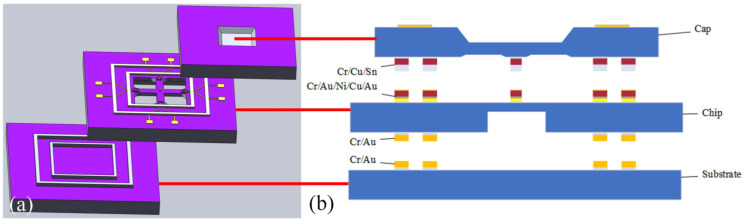
Schematic of test structures. (**a**) Graphene sensor structure (core crossbeam dimensions: 900 μm × 200 μm × 90 μm). (**b**) Bonding structure.

The schematic of test structures is shown in Figure 1. The area of silicon cap is 5.2 × 5.2 mm^2^, which consists of two sealing rings, round bump, and pressure cavity. The core chip and substrate were the same area of 6.5 × 6.5 mm^2^. There are only sealing rings in the substrate, and the chip is composed of a cruciform frame structure, wiring, Au electrodes, sealing rings, round bump, and sensitive unit. The silicon cap was firstly bonded to the core chip through Cu–Sn bonding, and the chip was then bonded to substrate via Au–Au bonding. Sealing ring is designed to protect the graphene, while round bump is transmitted displacement. As the pressure acts on the pressure cavity to deform the silicon cap, the root of crossbeam generates strain, resulting in the deformation of the graphene at the corresponding position. Finally, the measurement resistance of graphene changes.

### 2.2. Vehicle Fabrication

**Figure 2:**
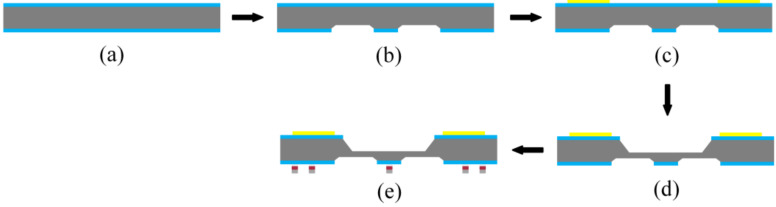
Schematic of the process for fabrication of the silicon cap. (**a**) Depositing the double-side SiNx. (**b**) Etching the front pressure cavity. (**c**) Sputtering the back metal ring. (**d**) Etching the back pressure cavity. (**e**) Evaporating the bump and sealing rings.

**Figure 3:**
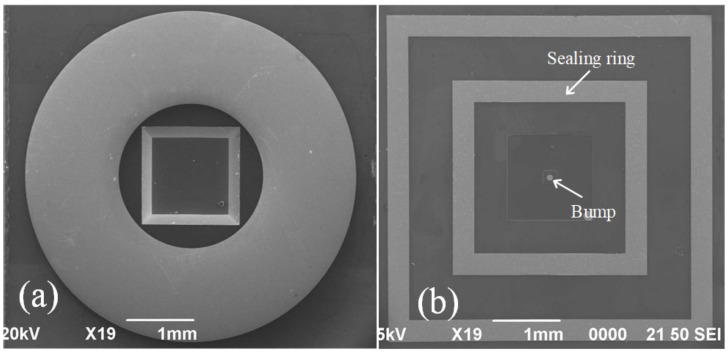
SEM images of the silicon cap. (**a**) Backside. (**b**) Frontside.

The cap fabrication process is shown in Figure 2. A double-sided 110 nm-thick SiN_x_ film was first deposited on a new 250 µm-thick silicon wafer following the low-pressure chemical deposition (LPCVD) method (Figure 2a). It protected the area from etch during the process of KOH wet etching. The inductively reactive ion etching (RIE) process was carried out at an etching rate of 50 nm/min, the cavity was characterized by a plane size of 1314 µm × 1314 µm with a center retaining 200 µm × 200 μm convex plate. It was developed on the front face of silicon. Following this, KOH solution (48%) was used to corrode the cavity with a depth of 12 µm at 85 °C, and etching rate was maintained at 1 µm/min (Figure 2b). The LPCVD method was followed to deposit 110 nm SiN_x_ films to protect the frontal pressure cavity. The Cr–Au layers with thicknesses of 50 and 300 nm were prepared following the magnetron sputtering (MS150, FHR, Inc.) for connection package shell (Figure 2c). Similar to the frontal pressure cavity, RIE and KOH solution were used to etch back pressure cavity, the size of the cavity was 970 µm × 970 µm with the depth of 220 µm (Figure 2d). Finally, The Cr–Cu–Sn layers with thicknesses of 50, 2000, and 2000 nm, respectively, were deposited to form the final bonding structures of the bumps and sealing ring. The equipment was subjected to the process of thermal evaporation (Figure 2e). A scanning electron microscopy (SEM) image of the front side of the final silicon cap is shown in Figure 3b, and the backside is shown in Figure 3a.

**Figure 4:**
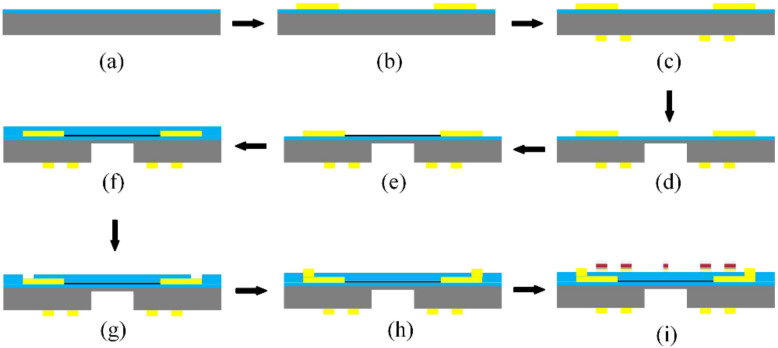
Schematic of the process for fabrication of the core chip. (**a**) Depositing the front SiN_x_. (**b**) Sputtering the bottom electrodes. (**c**) Sputtering the back sealing rings. (**d**) Etching the back pressure cavity. (**e**) Transferring and patterning the graphene. (**f**) Depositing the insulation layer. (**g**) Etching the insulation layer on electrode. (**h**) Sputtering the top electrodes. (**i**) Evaporating the front bump and sealing rings.

**Figure 5:**
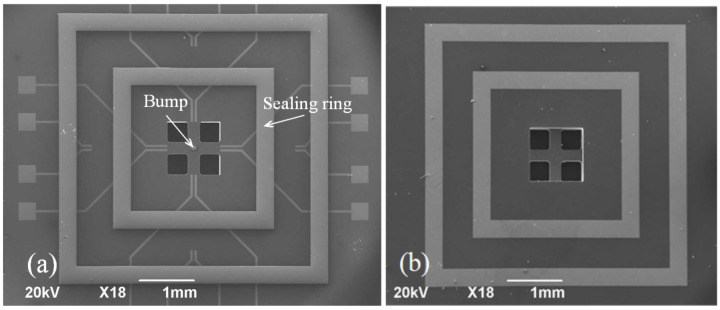
SEM images of the core chip. (**a**) Frontside. (**b**) Backside.

The core chip fabrication process is shown in Figure 4. Firstly, a 200 nm-thick SiN_x_ layer was deposited on one side as an insulation layer following plasma-enhanced chemical vapor deposition (PECVD) (Figure 4a). The Cr–Au layers with thicknesses of 15, 25 nm were deposited to form the bottom electrode (Figure 4b). Following this, the substrate and core chip sealing ring were simultaneously fabricated because the same pattern, and the substrate has been prepared in advance of the insulation layer. The Cr–Au layers with thicknesses of 50, 300 nm were deposited to form the back sealing rings by magnetron sputtering (Figure 4c). A 310-µm-deep pressure cavity with planar dimensions of 847 µm × 847 µm was then created at the back of silicon following the inductively coupled plasma (ICP) technique. The etching rate was maintained at 9.6 µm/min (Figure 4d). The next step involves the preparation of the key force-sensitive unit. This is graphically realized by the O_2_ plasma etching process. The process was conducted using wet transferred single layer graphene as the force-sensitive material (Figure 4e). Subsequently, SiN_x_ with a thickness of 200 nm was deposited formed an insulation layer to protect the surface wiring by PECVD. It is preventing the electrode from conducting with the sealing ring. On the one hand, SiN_x_ can effectively avoid graphene doping with water or air. On the other hand, SiN_x_ produced n-type doping of graphene and improves the stability of sensor [29] (Figure 4f). Meanwhile, the RIE method was used to etch the insulation layer on the electrode to ensure that there are no problems with the electrical connection (Figure 4g). The Cr–Au layers with thicknesses of 25 and 100 nm were deposited to form the top electrode by magnetron sputtering, graphene was located between the bottom electrode and the top electrode, which can ensure a low metal–graphene contact resistance (Figure 4h). The Cr–Au–Ni–Cu–Au layers with thicknesses of 50, 100, 100, 1500 and 3 nm, respectively, were deposited to form the final bonding structures of the bumps and sealing ring (Figure 4i). Finally, the ICP method was carried out to release the front cruciform frame structure. An SEM image of the front side of the core chip is shown in Figure 5a, and the backside is shown in Figure 5b.

### 2.3. Cu–Sn and Au–Au Bonding

**Figure 6:**
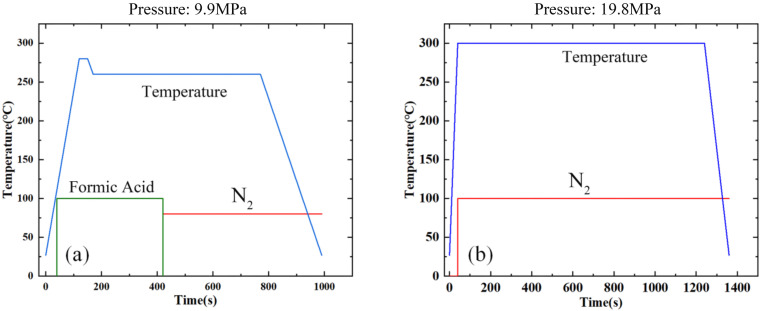
Bonding conditions. (**a**) Cu–Sn bonding. (**b**) Au–Au bonding.

Silicon cap and core chip surface needs to be pretreated for cleaning before bonding. The optimal pretreatment time set to be 120 s respectively as plasma power of 200 W and Ar (5% H_2_) gas flow rate of 200 sccm were fixed. After pretreatment, Cu–Sn bonding was performed with pressure of 9.9 MPa at 260 °C for 15 min in flip-chip bonder (FC150, SET, Inc.) [19]. The bonding conditions are shown in Figure 6a. The sample in an atmosphere of formic acid (cyan line) for 3 min, it was then subjected to an atmosphere of N_2_ (red line) until the end of the bonding process. To ensure the cleanliness of the subsequent Au–Au bonding surface, flip-chip bonder was cleaned before Cu–Sn bonding. Following this, Au–Au bonding was realized. Compared with Cu–Sn bonding, the pretreatment time is reduced to 60 s, other factors remain unchanged, and the corresponding process conditions for Au–Au bonding has increased to 300 °C, 20 min and 19.8 MPa [30,31]. The bonding conditions are shown in Figure 6b.

## 3. Results and Discussion

Four different tests were conducted to evaluate the bonding performance of the bonded device, including interface analysis, shear strength and hermeticity detection, high-temperature reliability, and static test.

### 3.1. Au Pretreatment Optimization

**Figure 7:**
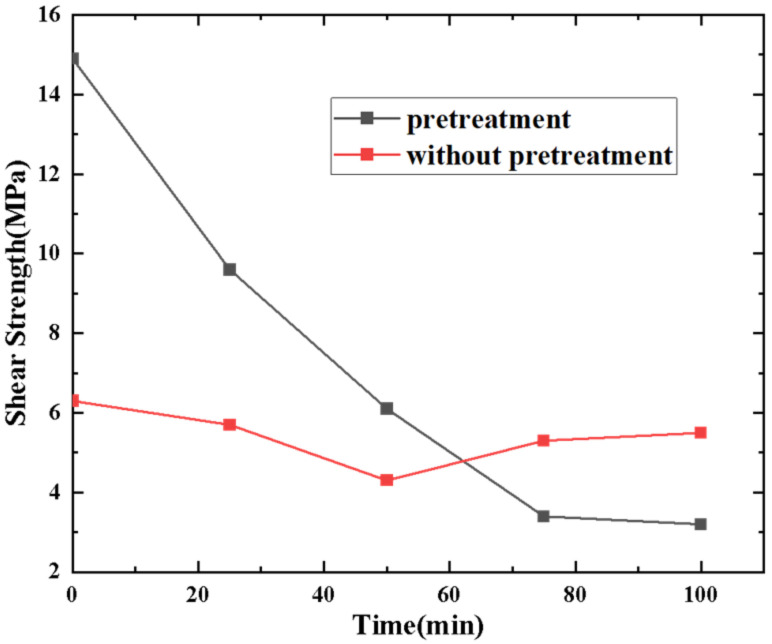
Bonding strength of samples with standing time.

**Figure 8:**
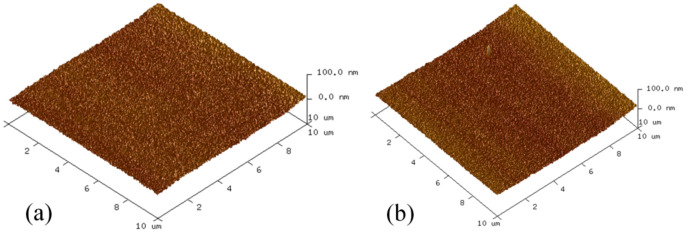
AFM images of Au surface. (**a**) Before pretreatment. (**b**) After pretreatment.

The pretreatment method followed before the formation of the Au–Au bonding can stimulate the Au surface activity, the Ar + H_2_ plasma treatment method could be used to increase the bonding strength. The strength achieved was higher than that achieved following the conventional Ar plasma treatment method [32]. To determine whether to conduct pretreatment, 10 samples were prepared, and divided into two groups; one group was pretreatment, and the other group was not. The result is shown in Figure 7. It can be seen that the shear strength decreases with increasing standing time (Time interval between plasma pretreatment and start of bonding). Meanwhile, the bonding strength of pretreatment sample is far greater than without pretreatment. Au surfaces roughness was characterized by atomic force tests (Figure 8a and Figure 8b, respectively). It was observed that the pretreatment surface was rougher than without pretreatment surface. The square roughness (Ra) increased from 2.6 nm to 4.7 nm.

### 3.2. Interfacial Analysis

**Figure 9:**
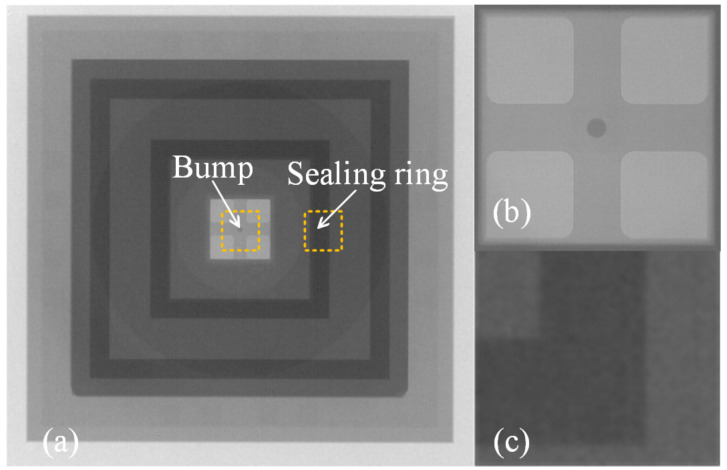
X-ray images. (**a**) Overall situation. (**b**) Round bump. (**c**) Sealing ring.

**Figure 10:**
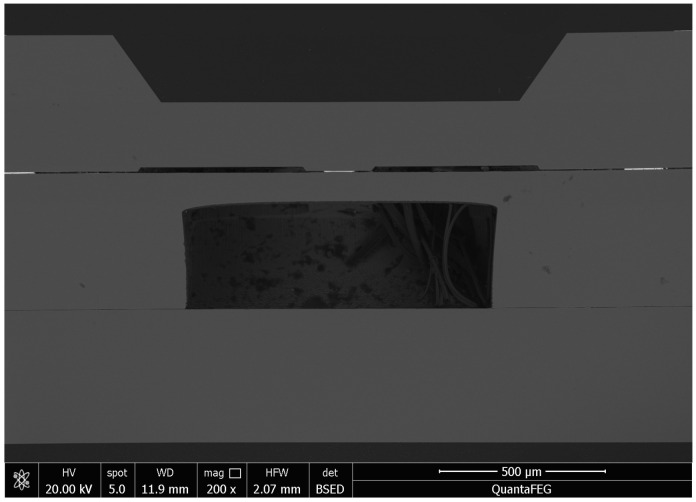
Dual bonding microscopic structure.

**Figure 11:**
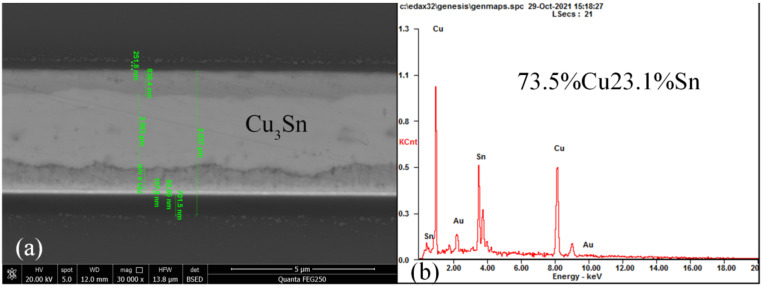
Cu–Sn bonding interfacial structure and composition. (**a**) Cross-sectional SEM image. (**b**) EDS spectrum.

**Figure 12:**
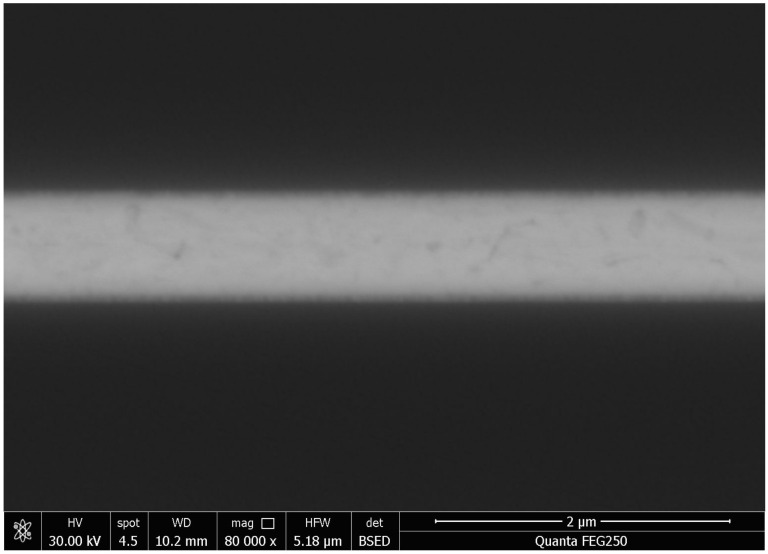
Au–Au bonding cross-sectional SEM image.

An X-ray image recorded for the dual-bonded microstructure is shown in Figure 9a. The images of the corresponding round bumps and sealing ring are shown in Figure 9b,c, respectively. Overflow of Sn was not observed for Cu–Sn bonding. Au did not melt for Au–Au bonding, and therefore no obvious spillover effect was observed. A cross-sectional SEM image of the microstructure of a distinct three-layers bonding interface is shown in Figure 10. The upper sealing ring and round bump could be seen, while the lower Au–Au bonding was not apparent. This can be attributed to the fact that Au units were thinner. A cross-sectional SEM image of Cu–Sn bonding interfacial microstructure is shown in Figure 11a. The results obtained using the energy-dispersive spectroscopy (EDS) (Genesis, EDAX, Inc.) technique are presented in Figure 11b. It shows the bonding interface can be divided into three layers. The upper and lower layers contain Cu (thicknesses of 0.83 and 0.79 µm, respectively). The middle layer is intermetallic compound (IMC) layer (with no obvious cracks and gaps) grown during the process of solid–liquid diffusion with thickness of 2.56 µm. Results from EDS analysis revealed that the ratio of copper atoms to Sn atoms was approximately 3:1. This intermediate layer was determined to be Cu_3_Sn. The densities of Cu_3_Sn and Cu were similar. When Cu_3_Sn grows on the Cu surface, the extent of volume change is small. This results in the generation of dense structures, reasonable metal structures, and few Cu_6_Sn_5_. Kirkendall effect can be avoided [33]. Au–Au bonding interface is shown in Figure 12. Obvious cracks and gaps in the middle metal layer were not observed. The interface was relatively flat, and width of metal layer did not change significantly. Thus, an excellent interconnecting interface was formed.

### 3.3. Shear Strength and Hermeticity Detection

**Figure 13:**
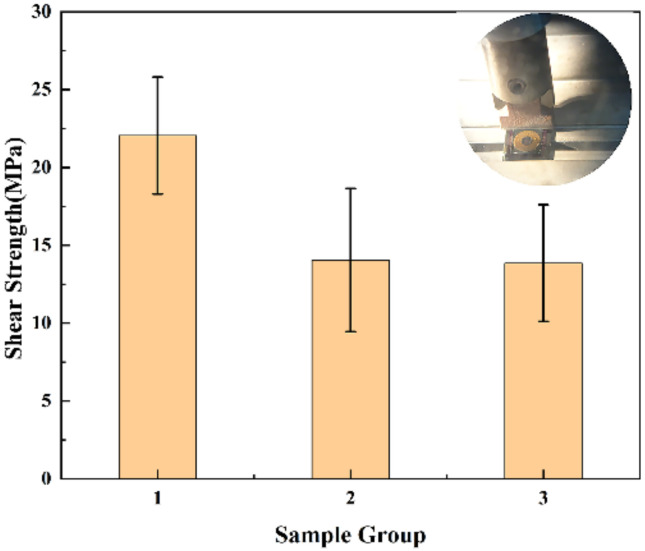
Bonding strength of the Cu–Sn bonding, Au–Au bonding, and dual bonding.

**Figure 14:**
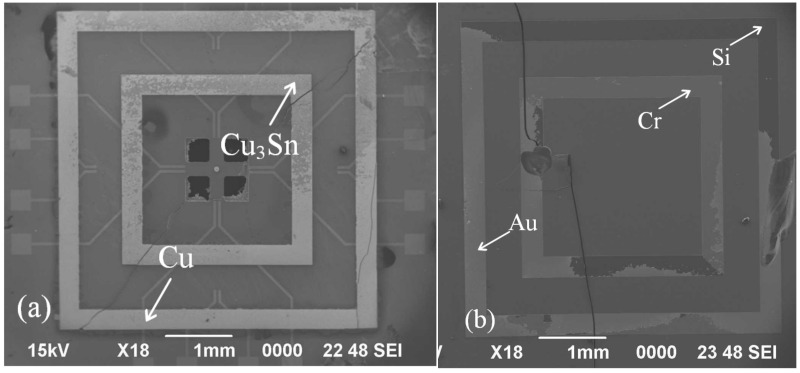
SEM images of the fracture surfaces for bonded device. (**a**) Cu–Sn bonding. (**b**) Au–Au bonding.

The shear strength was qualitatively assessed by conducting shear experiments using shear force tester (DAGE4000, Nordson DAGE, Inc.). The overall shear strengths of Cu–Sn, Au–Au, and dual-bonding devices were tested. The results are shown in Figure 13. A total of 15 bonded samples were tested and divided into three groups. The shear strength of Cu–Sn, Au–Au, and dual bonded were found to be in the range of 19.4–24.7 MPa, 10.8–17.3 MPa, and 11.2–16.5 MPa, the average shear strength was 22.5 MPa, 14.8 MPa, and 14.3 MPa. Shear test showed that shear strength of Cu–Sn bonding was higher than that of Au–Au bonding. Thus, the dual-bonding fracture finally occurred at Au–Au bonding. For Cu–Sn bonding, the fracture usually occurs in the Cu_3_Sn layer. This indicates that Cu_3_Sn is the weakest layer formed during Cu–Sn bonding. However, Au–Au bonding fracture partially occurs at the original interface present between Au and Au, and the other part occurs at the interface between Cr and Si or Cr and Au. SEM images of the fracture surfaces of the bonded samples are shown in Figure 14.

High-hermeticity packaging can provide both a pressure reference value and sensitive membrane protection for graphene pressure sensors. According to the MIL-STD-883K method 1014.15, with a volume of 0.25 mm^3^, the leak rate limit corresponding to the hermetic cavity was 5 × 10^−3^ Pa·cm^3^/s. Five samples were first placed under an atmosphere of helium at a pressure of 0.4 MPa over a period of 3 h. The helium that became attached to the surface of the samples was removed under a flow of nitrogen, and the leak rate was then measured using a helium mass spectrometer leak detector. The results are presented in Table 1.

For the bonding samples, the leakage rate was in the range of 1.46 × 10^−4^–2.47 × 10^−4^ Pa·cm^3^/s. The average value was 1.72 × 10^−4^ Pa·cm^3^/s which is significantly lower than the leak rate rejection limit.

### 3.4. High-Temperature Reliability

**Figure 15:**
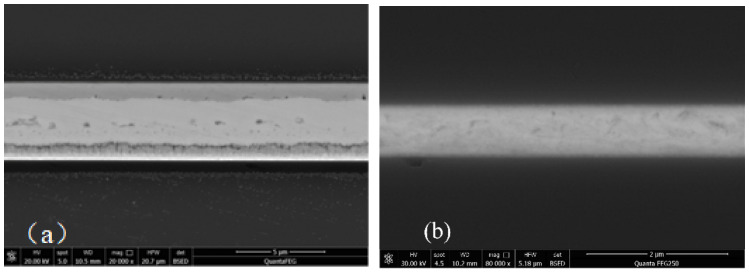
SEM images of the stored interfacial structure. (**a**) Cu–Sn bonding. (**b**) Au–Au bonding.

To verify the high-temperature reliability, HTS at 350 °C for 10 h was arranged. A cross-sectional SEM image of the stored interfacial microstructure for Cu–Sn bonding was recorded which is shown in Figure 15a. Some small voids were present, but obvious defects in the bonded interface were absent. This indicates that the quality of the bonding interface is almost unaffected by the HTS test. However, this may influence shear strength and hermeticity. The Au–Au bonding is shown in Figure 15b; the interface included a smooth interface with no void looks like that before HTS.

Then, five dual-bonding samples were tested, and the shear strength was found to be in the range of 11.5–16.3 MPa. The average shear strength was 13.5 MPa. The five samples in Table 1 are still used for hermeticity experiment after HTS. The results are shown in Table 2, compared with the data in Table 1. It is found that the leakage rate of each sample has increased to some extent, which presumably related to the formation of voids observed at the Cu–Sn interface before. The average value was 2.1 × 10^−4^ Pa cm^3^/s, but the overall change of shear strength and hermeticity is small. These results indicate that the dual-bonding structure can be effectively used in high-temperature environments. The bonding interface exhibits excellent performance even after the samples were subjected to conditions of the HTS test.

### 3.5. Static Test

**Figure 16:**
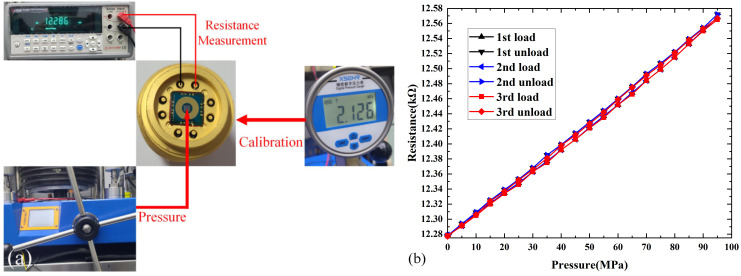
Electromechanical characteristics of the pressure sensor. (**a**) The schematic diagram for graphene pressure sensor measurement. (**b**) Relationships between the pressure and resistance for three cycles.

A multimeter was first used to test whether the resistance of graphene was still present after HTS. The results showed a slight decrease in the resistance of graphene compared with before HTS. This decrease can be attributed to the different thermal matching of graphene and metal electrodes at high temperatures. Then we used a piston manometer to pressurize graphene devices. The experimental arrangement is connected the precision digital pressure gauge to a piston manometer which can record the real time pressure of piston manometer. The change in the resistance value of the graphene sensor was recorded by digital multimeter. The output resistance versus the pressure curve is shown in Figure 16. The graph includes three cycle tests, with each cycle test including pressurization and relief process. The output resistance response showed excellent repeatability and stability. The sensitivity of the pressure sensor is expressed using the following equation:

S = ∆R/∆P(1)

where ∆R is the output resistance variation value, and ∆P is the pressure variation value. The average sensitivity of the pressure sensor is 3.11 Ω/MPa. Because there is no circuit in this case, the advantage of device sensitivity cannot be exploited. Relevant circuits are then designed to improve the sensitivity of the output response.

## 4. Conclusions

Hermetic packaging for a high-temperature graphene pressure sensor was realized based on the dual bonding of Cu–Sn and Au–Au. The dual-bonding performance was evaluated through various experiments. The bonding interface of Cu–Sn had no Sn overflow and transformed into a stable Cu–Cu_3_Sn–Cu structure, and Au–Au bonding included a smooth interface with no void. Shear test showed that shear strength of Cu–Sn bonding was higher than that of Au–Au bonding. Thus, it is reasonable that the final fracture of the dual-bonding samples occurs mostly at the Au–Au bonding. Hermeticity of the dual bonding was one order of magnitude less than standard leakage rate. After HTS test at 350 °C for 10 h, the dual-bonding performance also revealed no obvious change. The output resistance response exhibited considerable sensitivity and outstanding repeatability through three cycles of static pressure test, which was mainly attributed to the excellent sealing protection. In conclusion, dual bonding of Cu–Sn and Au–Au would be suitable for hermetic packaging in a particular condition and further promote the development of high-temperature graphene pressure sensor.

## Figures and Tables

**Table 1:** Hermeticity tests (×10^−4^ Pa·cm^3^/s).

**As bonded**	**Sample 1**	**Sample 2**	**Sample 3**	**Sample 4**	**Sample 5**
	1.53	1.48	1.66	2.47	1.46

**Table 2:** Hermeticity tests (×10^−4^ Pa·cm^3^/s).

**Stored**	**Sample 1**	**Sample 2**	**Sample 3**	**Sample 4**	**Sample 5**
	1.65	1.87	2.39	2.86	1.73

## Data Availability

Not applicable.
